# Multiscale screening and identifying specific targets for artesunate in suppressing bladder cancer

**DOI:** 10.3389/fphar.2025.1584502

**Published:** 2025-04-15

**Authors:** Yi Yuan, Guangdi Chu, Qingyue Ma, Zhijuan Liang, Ye Liang, Haitao Niu

**Affiliations:** ^1^ Department of Urology, The Affiliated Hospital of Qingdao University, Qingdao, China; ^2^ Department of Ophthalmology, The Affiliated Hospital of Qingdao University, Qingdao, China; ^3^ Key Laboratory, Department of Urology and Andrology, The Affiliated Hospital of Qingdao University, Qingdao, China

**Keywords:** artesunate, anti-tumor mechanism, bladder cancer, network pharmacology, molecular docking

## Abstract

**Background:**

Bladder cancer (BLCA) is a highly aggressive urinary malignancy with high mortality in advanced stages, posing a significant health risk. Artesunate (ART), a derivative of artemisinin, has been demonstrated with potent anti-tumor activity in some studies, yet its specific targets for BLCA and the molecular mechanisms have not been fully elucidated.

**Purpose:**

This study screened potential targets of ART against BLCA through network pharmacology, followed by molecular docking simulations and experimental validation *in vitro* and *in vivo* to elucidate the underlying mechanisms.

**Methods:**

This study identified the critical targets of BLCA and ART by employing multiscale screening from public databases, and a protein-protein interaction (PPI) network was constructed. Molecular docking simulations confirmed the stable binding of ART to the identified tumor-related targets promoting BLCA progression. These computational findings were further validated through experiments *in vivo* and *in vitro*, ensuring robust and reliable results.

**Results:**

Based on network pharmacology analysis, the effects of ART on BLCA were multifaceted. Molecular docking simulations confirmed the binding stability of ART with core targets. The experiments *in vitro* proved that ART could inhibit BLCA cell proliferation and migration by downregulating the expression of BCL-2, inducing Caspase 3-mediated apoptosis, resulting in cell cycle arrest and suppressing the PI3K/Akt/mTOR classical pathway involved in BLCA growth and metabolism. Studies *in vivo* also confirmed that ART had significant anti-tumor effects with minimal side effects.

**Conclusion:**

This study identified the mechanism by which ART inhibited BLCA through multiple specific targets, revealing its potential anti-cancer pathways and laying the foundation for the clinical application of traditional Chinese medicine in BLCA therapy.

## Graphical Abstract

**Figure:**
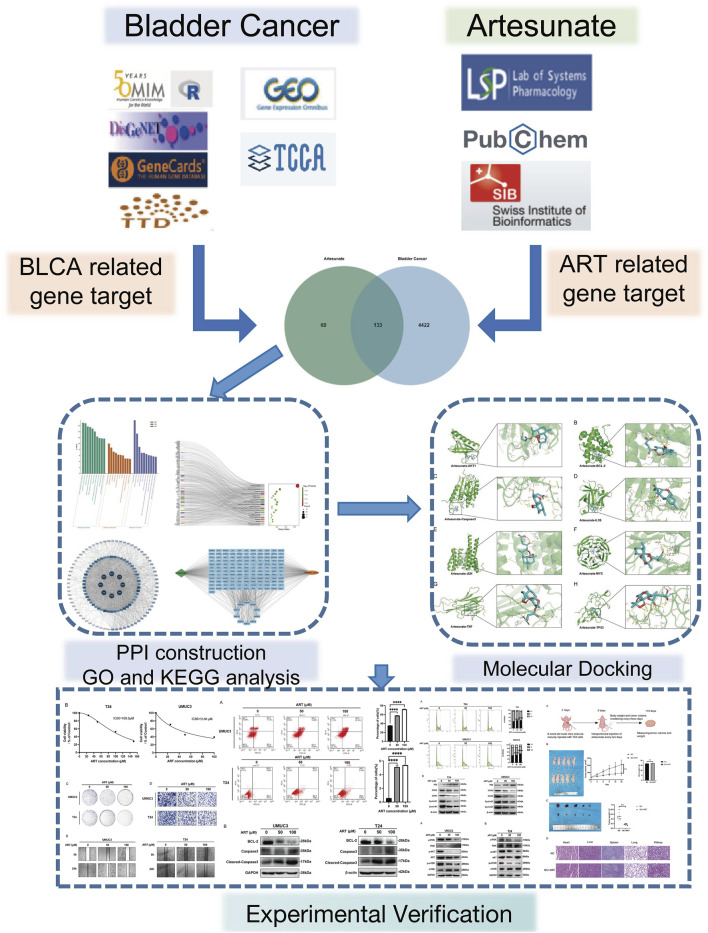
Flow of the research.

## 1 Introduction

Bladder cancer (BLCA) is the most common malignancy of the urinary system. According to statistics, in 2024, the United States has 83,190 new cases of BLCA, with 16,840 deaths attributed to the disease (Siegel et al., 2024). At initial diagnosis, approximately 70%–75% of patients present with non-muscle-invasive bladder cancer (NMIBC), 20%–25% with muscle-invasive bladder cancer (MIBC) and about 5% have distant metastases. Despite the improvements in patient survival brought about by current treatment methods, including surgical resection, intravesical instillation therapy and recently developed immunotherapy, several challenges remain, such as significant side effects, poor patient tolerance and limited efficacy. Therefore, it is crucial to develop novel treatment strategies, particularly those that are highly effective and have fewer side effects, to improve the survival rate and quality of life of BLCA patients (Witjes et al., 2021; Hermans et al., 2018).

Network pharmacology, as an emerging research tool, integrates systems biology, multidimensional data analysis and bioinformatics to support drug development and precision medicine. Unlike the traditional “single drug-single target” research model, network pharmacology systematically identifies potential drug targets, deeply analyzes their complex biological mechanisms and facilitates the development of personalized treatment strategies. Its core principle is to use computer simulations and huge data analysis to construct a “drug-target-disease” network, comprehensively elucidating the multi-target action mechanisms of drugs, predicting potential therapeutic targets and exploring possible drug combination strategies (Zhao et al., 2023). In the study of natural compounds, network pharmacology exhibits unique advantages. Natural compounds often possess complex chemical structures and multiple biological activities, allowing them to act on several signaling pathways and molecular targets simultaneously, thereby achieving synergistic anti-cancer effects. Therefore, network pharmacology is particularly important in elucidating the multi-target anti-cancer mechanisms of natural compounds. Compared to the traditional single-target drug development model, network pharmacology systematically constructs and analyzes the multidimensional biological interaction networks of natural compounds, providing crucial theoretical support for uncovering their anti-cancer mechanisms, identifying key targets and optimizing precision treatment strategies. This approach not only facilitates a comprehensive understanding of the pharmacological actions of natural compounds but also promotes the development and clinical translation of novel anti-cancer drugs.

Artesunate (ART) is a semi-synthetic derivative of artemisinin, which is derived from *Artemisia annua*. The discovery of artemisinin was awarded the Nobel Prize in Physiology or Medicine in 2015 for its outstanding anti-malarial activity. Recent studies have demonstrated that ART exhibited significant anti-cancer activity in various malignancies by blocking tumor-associated signaling pathways through multiple mechanisms (Efferth, 2017). For example, ART enhanced the radiosensitivity of esophageal cancer cells by inhibiting DNA damage repair (Fei et al., 2018); induced ROS-dependent apoptosis and ferroptosis in non-small cell lung cancer (Zhang et al., 2021); and targeted oral squamous cell carcinoma by inhibiting the AKT/AMPK/mTOR pathway (Xiao et al., 2020). Additionally, relevant studies *in vitro* have shown that ART, when combined with gemcitabine, can reverse gemcitabine resistance in pancreatic cancer cells (Yao et al., 2022). In terms of clinical applications, ART has made significant progress, demonstrating satisfactory efficacy and safety. For instance, in an open-label phase I clinical trial (ARTIC M33/2), 13 patients with metastatic breast cancer continued receiving ART as adjunct therapy after the trial ended. The results showed enhanced therapeutic efficacy, well tolerance and fewer side effects (von Hagens et al., 2019). Furthermore, 28 patients who received intravaginal ART implants for the treatment of CIN2/3 (cervical intraepithelial neoplasia grade 2/3) also exhibited favorable safety and tolerance (Trimble et al., 2020).

Currently, research on ART in BLCA primarily focuses on its mechanisms of drug resistance. Studies have found that in BLCA, ART can induce cell cycle arrest, trigger mitochondrial dysfunction, activate autophagy and mediate apoptosis in cisplatin-resistant BLCA cells (Li et al., 2024). Additionally, ART can generate reactive oxygen species (ROS), causing intracellular redox imbalance and making it a potential candidate for combination therapy with cisplatin in BLCA treatment (Chen et al., 2023). Furthermore, ART can promote tumor cell apoptosis by activating autophagy in BLCA cells (Zhou et al., 2020). However, the precise mechanism of ART in the treatment of BLCA remains to be fully elucidated. This study will integrate network pharmacology, molecular docking and experiments *in vitro* and *in vivo* to systematically explore the multi-target mechanisms of ART in BLCA, providing new insights and potential therapeutic strategies for precision treatment.

## 2 Methods

### 2.1 Cell culture and regents

Human BLCA cell lines T24 and UMUC3 were sourced from the Chinese Academy of Sciences Cell Bank. They were maintained in RPMI-1640 or DMEM with 10% FBS, incubated at 5% CO_2_ at 37°C. ART was purchased from MCE (HY-N0193; China). The drug powder was dissolved in DMSO (HY-Y0320; MCE; China) and then diluted in cell culture medium to the required concentration prior to use.

### 2.2 Network pharmacology analysis

A search for “artesunate” identified 201 targets, while “bladder cancer” yielded 4,555 targets. The intersection of these targets, along with transcriptome and clinical data from TCGA and GEO, was analyzed using Venny 2.1. Interactions between these targets were investigated using a PPI network built with STRING and represented visually through Cytoscape 3.10.1, with network centrality assessed using CytoNCA. GO and KEGG analyses were conducted using DAVID v6.8 and KEGG databases respectively, with results visualized using charts from the Bioinformatics platform.

### 2.3 Molecular docking

Gene IDs for core targets were obtained from UniProt and 3D protein structures were sourced from the PDB (https://www.rcsb.org/). The structure of ART was converted and optimized using Chem3D. Docking simulations were conducted with AutoDock Vina and the results were visualized using PyMOL. Binding energies were interpreted as follows: values below 0 kcal/mol indicated the ability of binding, below -5 kcal/mol indicated strong affinity and below -7 kcal/mol indicated extremely strong affinity (Ge et al., 2023).

### 2.4 MTT assay

Cells were seeded at a density of 2000 cells per well in a 96-well plate and allowed to adhere overnight. The following day, cells were treated with varying concentrations of ART and incubated for 24 h. Subsequently, 20 µL of MTT solution (5 mg/mL in PBS) was added to each well, and the plate was incubated for an additional 4 h at 37°C. After incubation, the medium containing MTT was carefully removed, and 150 µL of DMSO was added to dissolve the formazan crystals. The optical density (OD) was measured at 490 nm using enzyme-labeled instrument to quantify cell viability.

### 2.5 Colony formation

The BLCA cells T24 and UMUC3 were pretreated with varying concentrations of ART for 24 h. After pretreatment, 500 cells per well were seeded into six-well plates and cultured for 14 days to allow colony formation. At the end of the incubation period, colonies were fixed with 4% paraformaldehyde for 20 min and stained with 0.2% crystal violet solution for 30 min. Excess stain was rinsed off with distilled water, and the plates were air-dried before imaging and counting the colonies.

### 2.6 Cell migration

After pretreatment with varying concentrations of ART for 24 h, cells were resuspended in serum-free medium and seeded into the upper chambers of Transwell inserts with an 8 μm pore size. The lower chambers were filled with complete medium containing 10% serum as a chemoattractant. The plates were incubated in a 37°C cell incubator for 24 h. After incubation, migrated cells on the bottom surface of the membrane were fixed with 4% paraformaldehyde for 20 min and stained with 0.1% crystal violet for 30 min. The stained membranes were washed with distilled water, air-dried and non-migrated cells on the upper surface of the membrane were gently removed using a cotton swab. The membranes were then visualized under a microscope for analysis.

### 2.7 Flow cytometry

Cell cycle and apoptosis analyses were performed using flow cytometry with DNA-binding dyes and apoptosis markers. After pretreating the cells with ART at concentrations of 0, 50 and 100 μM for 24 h, the cells were harvested and stained with propidium iodide (PI) solution to assess cell cycle distribution. Additionally, apoptosis was evaluated by labeling the cells with Annexin V-FITC to detect early apoptosis and PI to label late apoptotic or dead cells. The samples were then analyzed using flow cytometry to determine the distribution of cells in the G0/G1, S and G2/M phases, and to quantify the populations of early and late apoptotic cells. Data analysis was conducted using Novepress software to assess the fluorescence intensity and determine the percentage of cells in each phase and apoptosis stage.

### 2.8 Western blot (WB)

Cells exposed to 0, 50 and 100 μM concentrations of ART for 48 h were lysed using RIPA buffer to ensure optimal protein extraction. The lysates were resolved by SDS-PAGE and transferred to PVDF membranes. Following overnight incubation at 4°C with primary antibodies, the membranes were treated with HRP-conjugated secondary antibodies for 1 h and protein signals were detected using chemiluminescence (ECL) (WBK1S0100; millipore; United States) Detailed information regarding the antibodies used can be found in (Supplementary Table 1).

### 2.9 Subcutaneous xenograft tumor model

Four-week-old female nude mice were purchased from Beijing Vital River Laboratory Animal Technology Co., Ltd. (Beijing; China). Following a 2-week acclimation period, tumor cells (1 × 10^7^ per mouse) were injected into the right axillae of the mice. Subsequently, the mice were randomly allocated into two groups. The ART group received 90 mg/kg of ART via intraperitoneal injection, while the control group was administered an equivalent volume of physiological saline. After 15 days of treatment, the mice were euthanized, and subcutaneous tumors were harvested and quantitatively measured. Moreover, tissues from the heart, liver, spleens, lungs and kidneys were collected for histopathological analysis using hematoxylin and eosin (HE) staining.

### 2.10 Statistical analysis

Data were processed and analyzed using Prism 8.0.1, employing one-way ANOVA and unpaired t-tests for statistical evaluation, and were presented as the mean of three independent samples. Statistical significance is indicated as *p < 0.05, **p < 0.01, ***p < 0.001, ****p < 0.0001.

## 3 Results

### 3.1 Potential targets of ART for BLCA treatment

**FIGURE 1:**
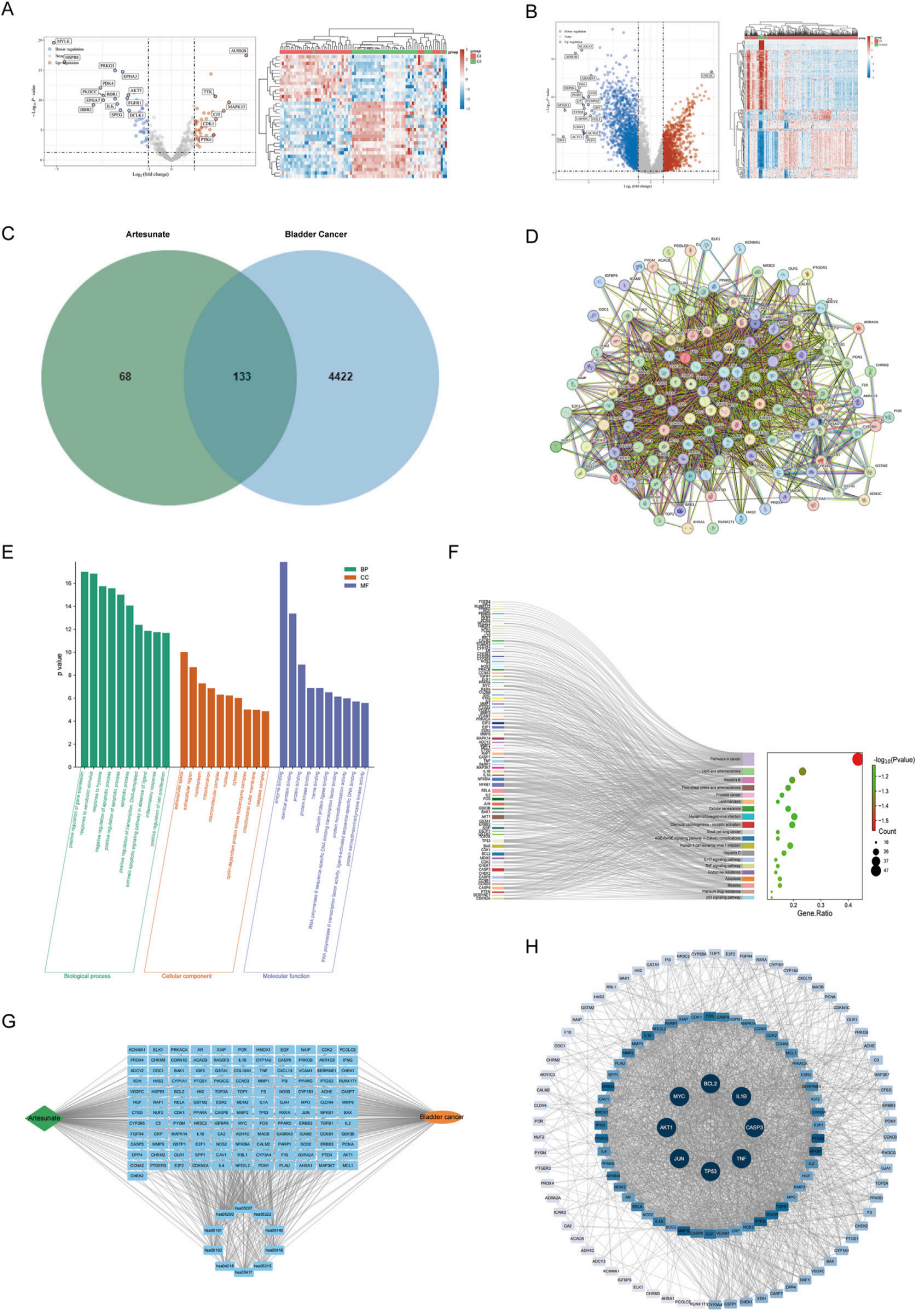
The potential targets of ART for treating BLCA. **(A)** Using data from the GEO database, a volcano plot and detailed expression profiles were generated to illustrate the DEGs between tumor tissues and adjacent normal tissues in BLCA. **(B)** Using data from the TCGA database, a volcano plot and detailed expression profiles were generated to illustrate the DEGs between tumor tissues and adjacent normal tissues in BLCA. **(C)** The Venn diagram of the intersecting targets of ART in BLCA. **(D)** PPI network based on STRING database analysis. **(E)** The Gene Ontology (GO) enrichment analysis identified the top 10 most significantly enriched terms in Cellular Component (CC), Biological Process (BP) and Molecular Function (MF) categories. **(F)** Top 20 enriched KEGG pathways. **(G)** Disease-Drug-Pathway-Targets network. **(H)** The core targets were presented in the form of a PPI network.

Conducted differential gene expression analysis between BLCA and adjacent tissues in the GEO and TCGA databases and visualized the results (Figures 1A,B). Performed an intersection analysis with BLCA-related gene targets retrieved from the OMIM, Gene-Cards, DisGeNET and TTD databases, resulting in 4,555 disease targets. By searching PubChem, TCMSP databases and predicting with Swiss Target Prediction, 201 potential targets of ART were identified. An intersection analysis of the 201 ART-related target genes and 4555 BLCA-related target genes resulted in 133 intersecting genes, which can be considered potential therapeutic candidate targets of ART for BLCA. The intersection results were displayed in Figure 1C. Following this, a drug-disease-target network of 133 genes was constructed using Cytoscape 3.10.1 software, demonstrating the interaction between ART and BLCA (Figure 1D). GO enrichment analysis identified 812 significant GO terms (*p* < 0.05), including 622 biological processes (BP), 66 cellular components (CC) and 124 molecular functions (MF) (Supplementary Table 2). The 10 most significantly enriched terms for each category were presented visually (Figure 1E), suggesting ART may counteract BLCA by regulating proteins and enzymes in these pathways. KEGG enrichment analysis revealed 175 significant pathways (*p* < 0.05) (Supplementary Table 3), with key tumor-related pathways like “cancer pathways,” “apoptosis” and “p53 signaling pathway” being prominent (Figure 1F). To investigate ART’s interaction with BLCA intersecting targets, 133 targets were analyzed using the STRING database and visualized with Cytoscape 3.10.1, creating a disease-drug-pathway-target network (Figure 1G). In the PPI network, darker and more central nodes indicated stronger interactions with central nodes representing core targets (AKT1, BCL-2, Caspase 3, IL1B, JUN, MYC, TNF, TP53) (Figure 1H). The analysis showed that these targets were involved in several tumor-related pathways (Supplementary Table 2), suggesting ART may affect BLCA by modulating these tumor-related processes.

According to these results, ART was expected to inhibit cancer by affecting specific physiological processes and key molecules in cancer. To confirm this hypothesis, additional molecular docking and experimental validation were next be performed.

### 3.2 The patterns of molecular docking simulation

**FIGURE 2:**
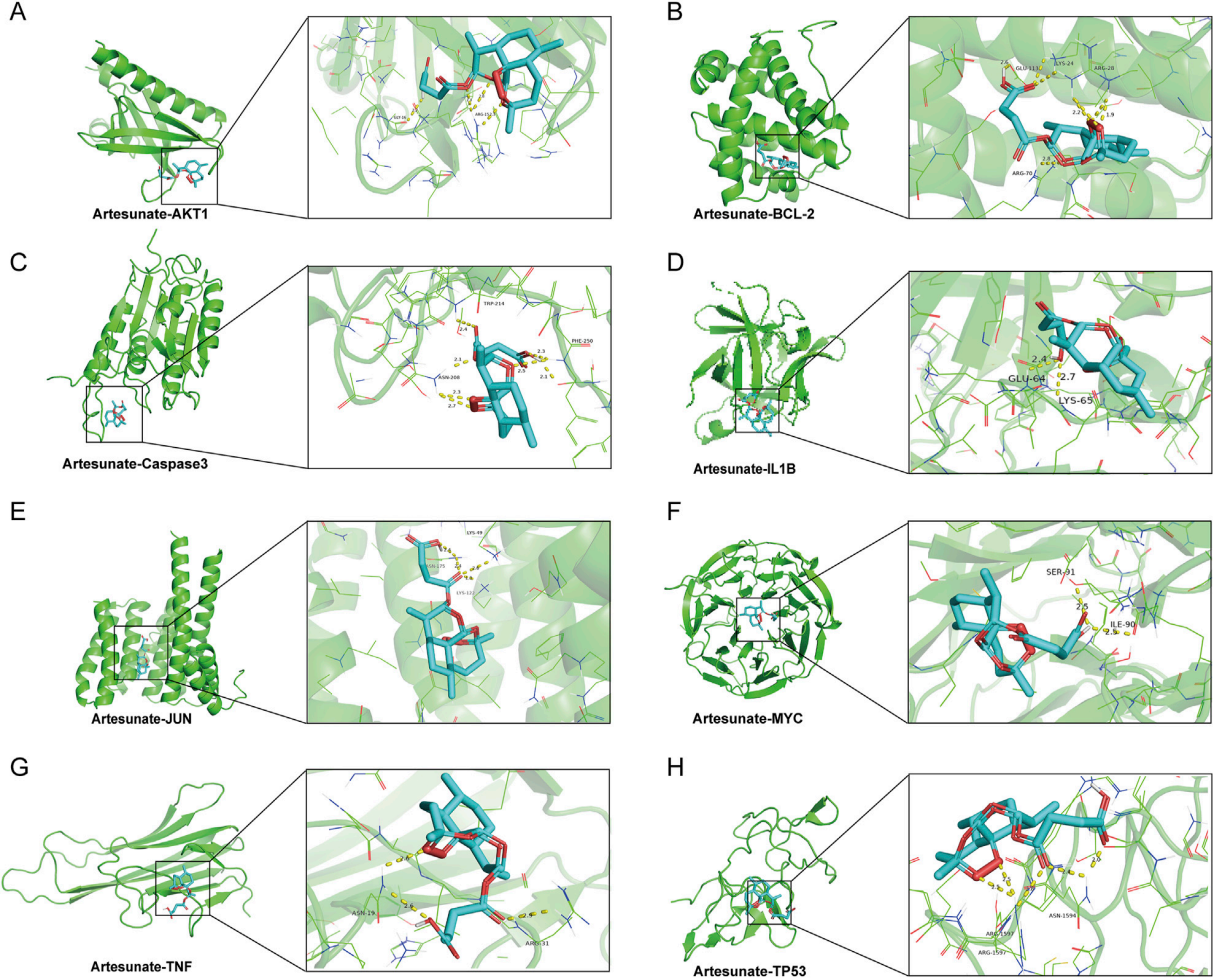
The patterns of molecular docking. **(A)** ART-AKT1. **(B)** ART-BCL-2. **(C)** ART-Caspase 3. **(D)** ART-IL1B. **(E)** ART-JUN. **(F)** ART-MYC. **(G)** ART-TNF. **(H)** ART-TP53.

To gain deeper insight into the role of ART in BLCA, this research further employed molecular docking simulations to comprehensively simulate the interactions between ART and the identified core targets. Specifically, ART formed multiple hydrogen bonds within the binding pockets of certain target proteins and interacted with key amino acid residues. These interactions may influence the conformational stability of target proteins and their functional roles in signaling pathways. Detailed information on binding affinity, hydrogen bond sites and bond lengths were provided in Table 1, while the visualized binding conformations of ART with core targets were presented in Figure 2. The docking results revealed that the binding affinities of ART with core targets ranged from −6.7 to −8.2 kcal/mol, indicating strong binding interactions between ART and these proteins.

**TABLE 1:** Detailed information on ART binding to core targets.

Targets	Binding energy (kcal/mol)	Hydrogen bond sites	Hydrogen bond length (Å)
AKT1	−7.0	GLY-16, ARG-15	2.2Å; 2.3Å, 2.8Å, 2.3Å
BCL-2	−6.7	GLU-113, LYS-24; ARG-28; ARG-70	2.6Å; 2.2Å; 1.9Å; 2.8Å
Caspase3	−7.1	ASN-208; TRP-214; PHE-250	2.3Å, 2.7Å, 2.1Å; 2.4Å; 2.1Å, 2.3Å, 2.5Å
IL1B	−6.8	GLU-64; LYS-65	2.4Å; 2.7Å
JUN	−7.0	LYS-122; ASN-175; LYS-49	1.9Å; 2.4Å; 2.6Å
MYC	−8.0	SER-91; ILE-90	2.5Å; 2.5Å
TNF	−6.7	ASN-19; ARG-31	2.6Å; 2.9Å
TP53	−8.2	ARG-1597; ASN-1594	2.5Å, 2.8Å; 2.0Å, 2.4Å

The above findings suggested that ART exhibited high-affinity binding to multiple key genes involved in tumor progression, playing a crucial role in tumor cell proliferation, migration and apoptosis. For instance, ART demonstrated a strong binding affinity with BCL-2, a critical anti-apoptotic protein that inhibited mitochondrial outer membrane permeability, thereby preventing cytochrome C release and suppressing apoptosis pathway activation (Iqbal et al., 2019). This suggested that ART may regulate BLCA cell apoptosis by targeting BCL-2. Moreover, ART exhibited strong binding interactions with Caspase 3 (CASP3), an essential executioner of apoptosis, implying that ART may mediate the activation of intrinsic or extrinsic apoptotic pathways, promoting programmed tumor cell death and exerting its anti-BLCA effects (Yang et al., 2017).

Additionally, ART displayed a strong binding affinity for TP53, a pivotal tumor suppressor gene encoding the p53 protein, which played a fundamental role in cell cycle regulation and tumor suppression (Ter Huurne et al., 2020). When DNA damage occurs, p53 facilitated either DNA repair or apoptosis initiation to prevent malignant transformation. The high binding affinity between ART and TP53 suggested that ART might exert its regulatory effects on BLCA by modulating cell cycle processes. Furthermore, ART exhibited strong interactions with AKT1, a key molecule in the PI3K/AKT/mTOR signaling pathway, which was widely activated in various malignancies and promotes tumor cell survival and invasive behaviors (Yu et al., 2022). These findings suggested that ART might exert anti-BLCA effects by disrupting this signaling pathway, thereby inhibiting tumor cell survival and malignant progression.

By integrating network pharmacology and molecular docking techniques, this research preliminarily identified multiple core targets and regulatory pathways through which ART might exert its effects in BLCA. These key targets not only play essential roles in tumorigenesis and progression but may also be closely associated with the unique biological characteristics of BLCA, providing valuable theoretical insights into the potential anti-BLCA mechanisms of ART. In the next part, we began to focus on validating these predictions through experiments *in vitro* and *in vivo*, and further elucidating the underlying molecular regulatory mechanisms.

### 3.3 ART inhibited BLCA proliferation and migration

**FIGURE 3:**
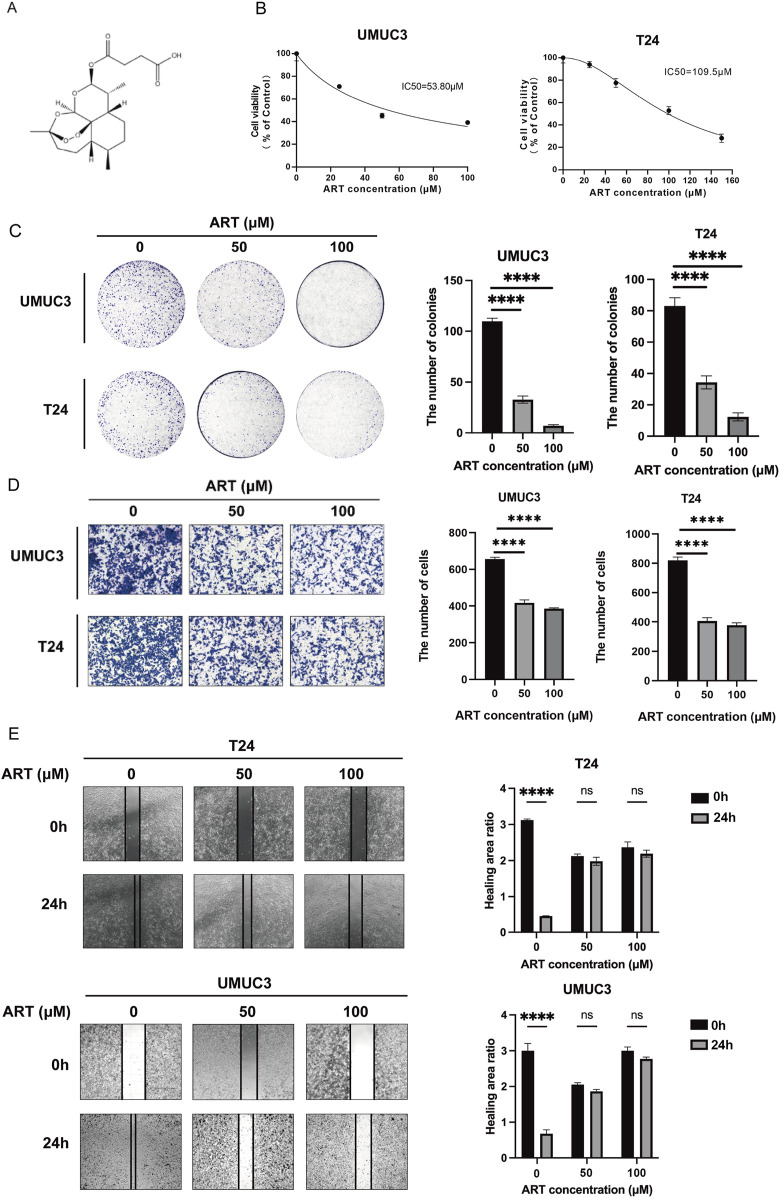
ART inhibited BLCA cells proliferation and migration. **(A)** The chemical structure of ART. **(B)** The MTT assays were used to measure the viability of UMUC3 and T24 cells. **(C)** ART reduced colony formation in UMUC3 and T24 cells in a dose-dependent manner. **(D)** ART decreased cell migration in both T24 and UMUC3 cells in a dose-dependent manner. **(E)** The wound healing assay assessed migration of UMUC3 and T24 cells after 24-h treatment with ART at concentrations of 0, 50 and 100 μM.

The chemical structure of ART was shown in Figure 3A. According to network pharmacology analysis, ART exhibited significant anti-tumor properties against BLCA. Initial evaluations of its efficacy were carried out using MTT, colony formation, wound healing and cell migration assays.

The effect of ART on BLCA cell growth was evaluated by treating cells with varying ART concentrations for 24 h and assessing viability with the MTT assay. The results showed that ART caused a dose-dependent decrease in cell viability, with IC50 values of 53.8 μM for UMUC3 cells and 109.5 μM for T24 cells (Figure 3B). Additionally, colony formation assays confirmed that ART reduced the ability of BLCA cells to form colonies in a dose-dependent manner (Figure 3C).

Controlling tumor migration is vital for mitigating its aggressive behavior. The wound healing and cell migration assays showed that ART effectively reduced the migration of T24 and UMUC3 cells in a dose-dependent manner (Figures 3D,E).

### 3.4 ART induced BLCA apoptosis

**FIGURE 4:**
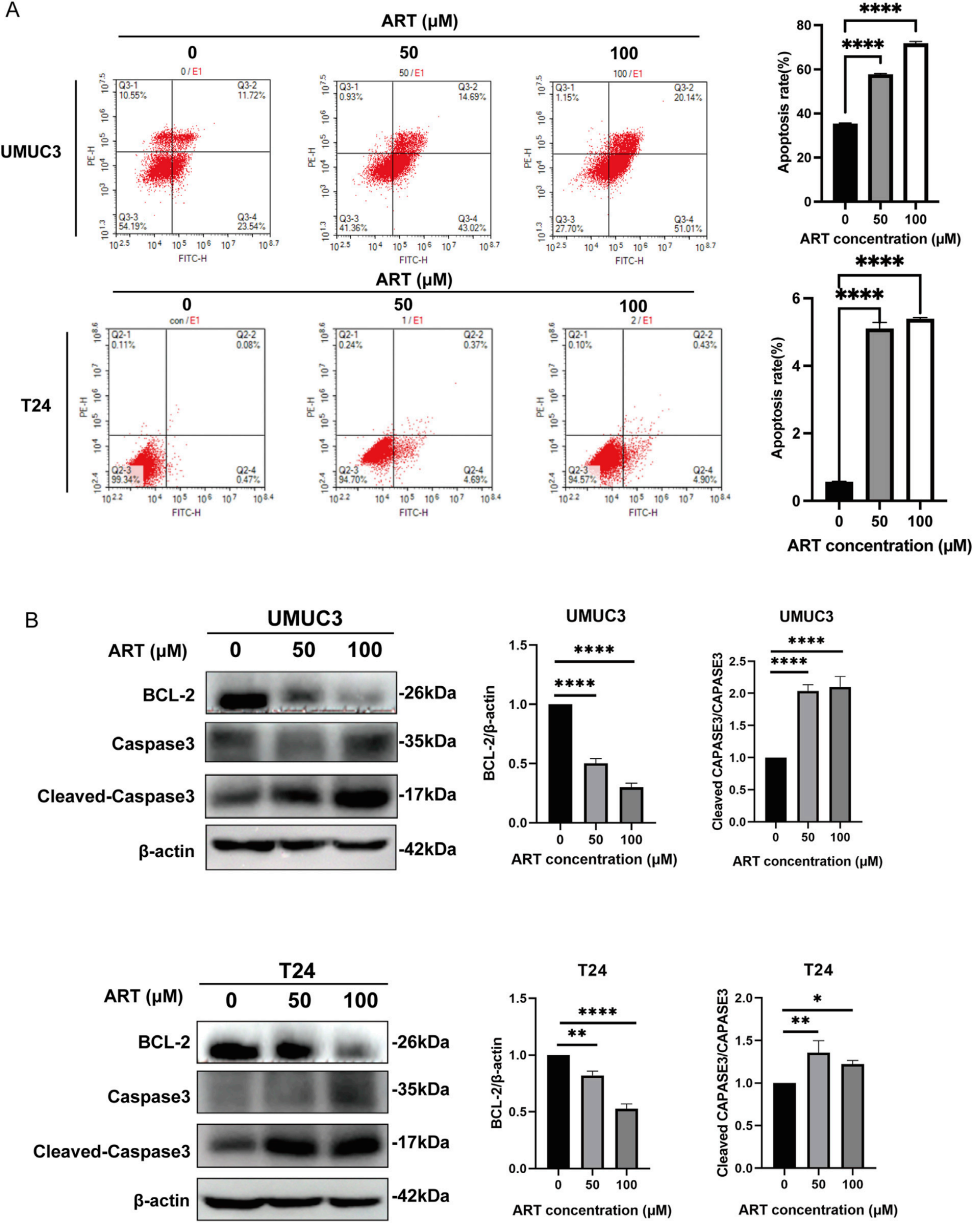
ART induced BLCA apoptosis. **(A)** The effects of ART treatment on apoptosis in UMUC3 and T24 cell lines were assessed through FACS analysis with Annexin V-FITC and PI staining. **(B)** Apoptosis-related proteins were examined via WB experiments.

Flow cytometry analysis revealed a substantial increase in apoptosis levels in BLCA cells with higher concentrations of ART (Figure 4A). WB experiments indicated that ART treatment reduced the expression of BCL-2 and increased the expression of Cleaved-Caspase 3/Caspase 3 in BLCA cells (Figure 4B). Cleaved-Caspase 3, a pivotal executioner of apoptosis, targets and cleaves various cellular substrates, ultimately driving cell death. Based on this, it can be strongly inferred that ART exerted its inhibitory effect on BLCA by inducing apoptosis via the Caspase pathway.

### 3.5 ART arrested BLCA cell cycle

**FIGURE 5:**
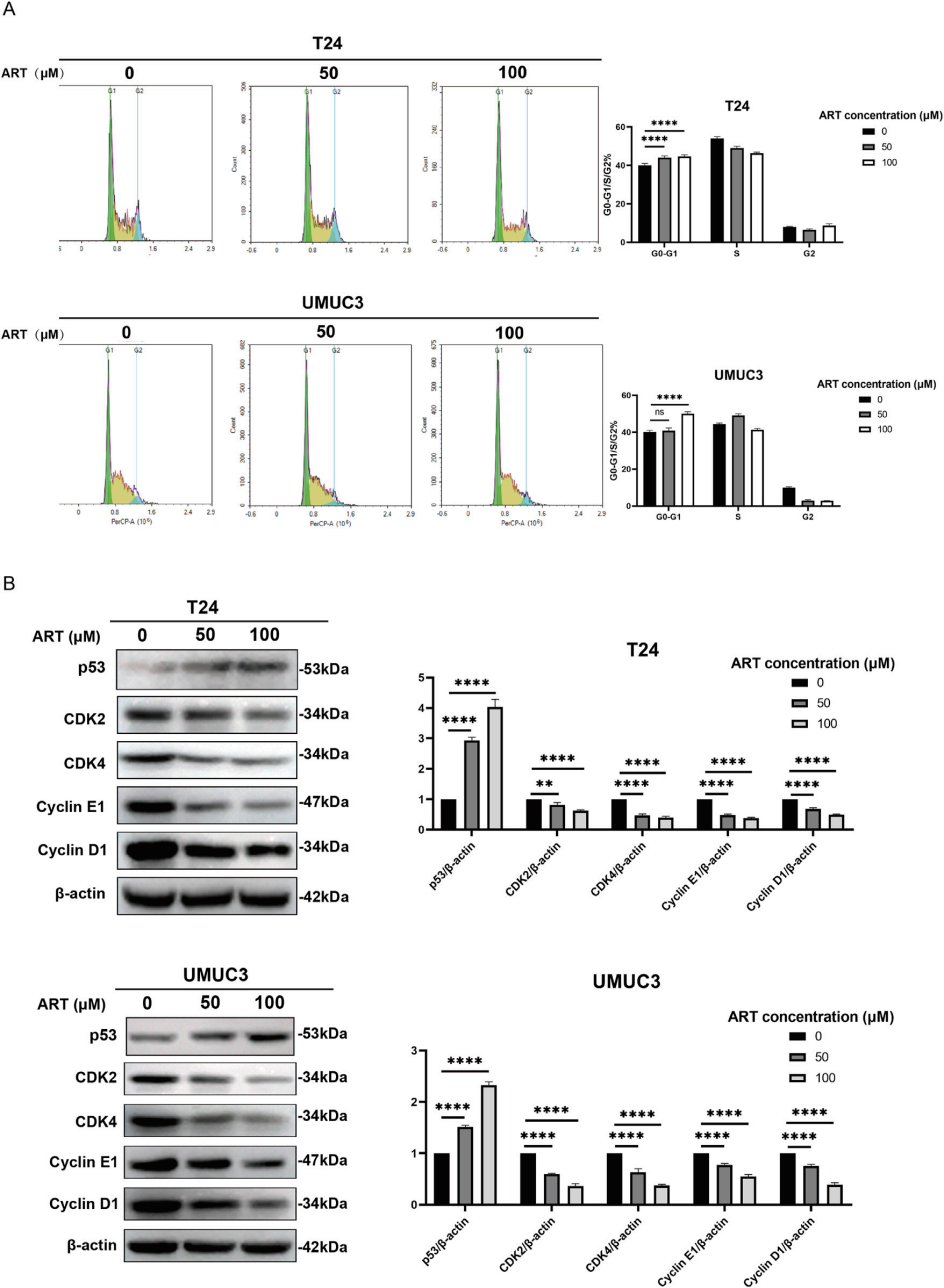
ART induced BLCA cell cycle arrest. **(A)** ART caused G1 phase arrest in T24 and UMUC3 cells, as shown by PI staining and flow cytometry. **(B)** WB experiments were used to measure the levels of proteins involved in cell cycle arrest.

Molecular docking simulation showed that ART bound strongly with p53 with a parameter of −8.2 kcal/mol. Given that p53 can halt the cell cycle at the G1 phase, we investigated whether ART had a similar effect on BLCA cells. After 24 h of ART treatment, flow cytometry revealed an increased proportion of cells in the G1 phase and a decreased proportion in the S phase, indicating that ART induced G1 phase arrest (Figure 5A).

Cyclin D-CDK4 and Cyclin E-CDK2 complexes are essential for regulating the transition from G1 to S phase. Cyclin D-CDK4 begins this transition by phosphorylating Rb protein early in G1, while Cyclin E-CDK2 completes it in late G1 and early S phase by further phosphorylating Rb and starting DNA replication. WB experiments showed that ART decreased the expression levels of CDK4, CDK2, Cyclin E1 and Cyclin D1 in BLCA cells in a dose-dependent manner (Figure 5B). Additionally, higher concentrations of ART led to increased levels of p53 protein, indicating that ART induced cell cycle arrest.

The findings indicated that ART affected cell cycle regulation in BLCA cells by blocking the transition from G1 to S phase, thereby contributing to its anti-BLCA effects.

### 3.6 ART inhibited the PI3K/AKT/mTOR signaling pathway in BLCA

**FIGURE 6:**
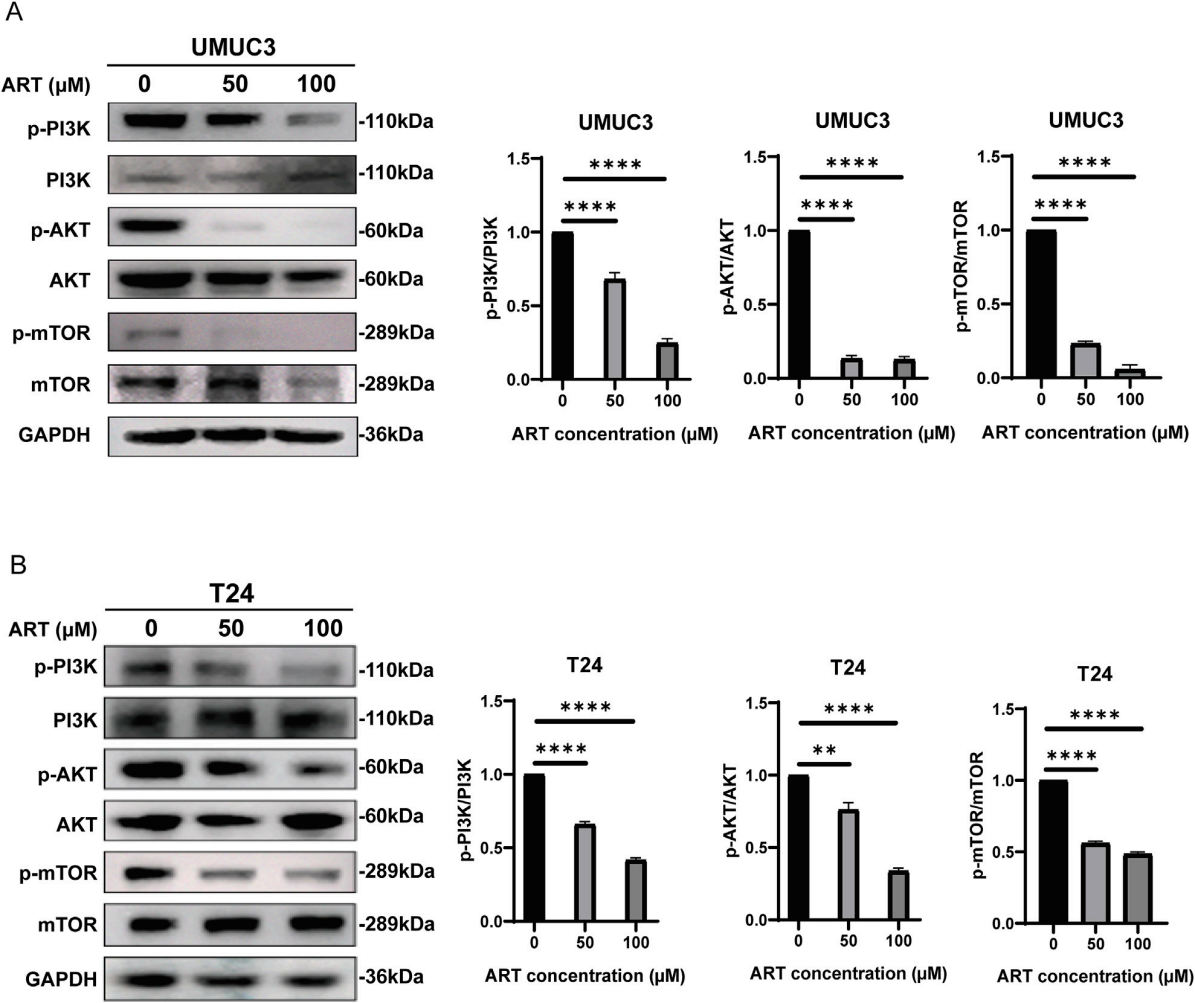
ART inhibited BLCA by targeting the PI3K/AKT/mTOR pathway. **(A,B)** WB were employed to assess the expression levels of PI3K, AKT and mTOR in BLCA cells treated with various concentrations of ART over a 24-h period.

The network pharmacology and molecular docking results indicated a strong interaction between ART and AKT. To assess whether ART disrupted the AKT signaling pathway, WB experiments were performed to reveal that ART treatment significantly reduced the levels of p-PI3K/PI3K, p-AKT/AKT and p-mTOR/mTOR compared to the control group (Figures 6A,B).

In summary, ART inhibited the activity of its corresponding signaling pathways by significantly reducing the phosphorylation levels of key proteins such as PI3K, AKT and mTOR in BLCA cells. This reduction in phosphorylation dampened the activity of the pathway, which was crucial for cell survival and proliferation, thereby potentially inhibiting cancer progression.

### 3.7 ART effectively exerted its inhibitory effects on BLCA *in vivo*

**FIGURE 7:**
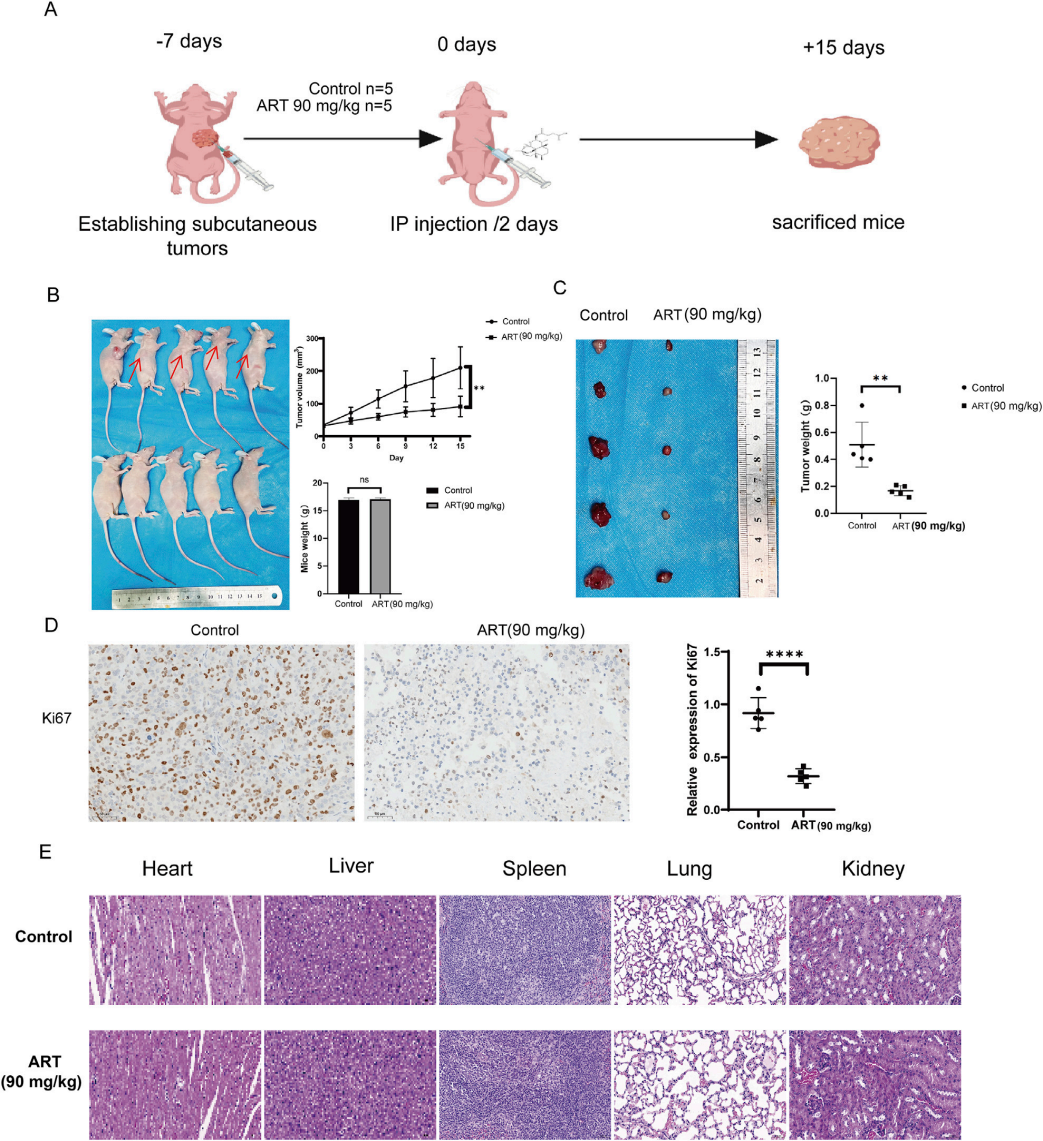
Assessment *in vivo* of the anti-tumor efficacy of ART in BLCA. **(A)** Six-week-old female nude mice were subcutaneously injected with 1 × 10^7^ T24 cells. One week later, the experimental group was intraperitoneally administered ART at a dose of 90 mg/kg, while the control group received an equivalent volume of saline. Injections were administered every 2 days, and tumor volume and body weight were monitored every 3 days. After 15 days of treatment, the mice were euthanized, and tumor volume and weight were subsequently assessed. The diagram was created with BioGDP.com (Jiang et al., 2025). **(B)** Tumor volume growth trajectory and body weight changes in nude mice. **(C)** Tumor size and mass in subcutaneously implanted nude mice. **(D)** Ki67 staining of subcutaneous tumors in nude mice. **(E)** HE staining of internal organs from the control and treatment groups of mice.

Studies *in vitro* unequivocally demonstrated the potent inhibitory effect of ART on BLCA cells. To further substantiate the pharmacological efficacy of ART, complementary experiments *in vivo* were conducted. As depicted in Figure 7A, a subcutaneous tumor model was established in nude mice, followed by intraperitoneal ART administration 7 days post-inoculation. Tumor size and weight were subsequently measured after 15 days of treatment. As illustrated in Figures 7B,C, the ART group displayed significant differences in tumor size and weight relative to the control group, suggesting that ART effectively suppressed further tumor growth. Moreover, no significant differences in body weight were observed between the two groups. IHC staining of subcutaneous tumors in nude mice revealed that the expression of Ki67, a proliferation marker, was significantly reduced in the ART group compared to the control group (Figure 7D). Furthermore, after harvesting the internal organs from both groups of nude mice and performing HE staining, no significant damage to the organs was observed in either group (Figure 7E). Additionally, no significant differences in body weight were noted between the two groups, suggesting that ART has minimal toxicity and side effects, with favorable drug safety.

## 4 Discussion

NMIBC is characterized by a high recurrence rate, with a subset of patients potentially progressing to MIBC or metastatic BLCA, thereby complicating treatment strategies (van Rhijn et al., 2009). At present, the management of BLCA predominantly relies on surgical resection, intravesical chemotherapy and immunotherapy (Patel et al., 2020). However, radical cystectomy can substantially impair a patient’s quality of life (Clements et al., 2022), while intravesical chemotherapy and immunotherapy are hindered by challenges such as drug resistance and adverse side effects, which limited their long-term efficacy (Han et al., 2020; Martins et al., 2019).

*Artemisia annua*, a traditional Chinese medicinal herb, had its main active component, artemisinin, widely used for its anti-malarial properties (Efferth, 2017). Recent studies have shown that both artemisinin and its semi-synthetic derivative, ART, held considerable promise for cancer treatment. ART, with its improved water solubility and bioavailability over artemisinin, has been demonstrated to exert anti-tumor effects by inducing apoptosis and inhibiting choroidal melanoma through angiogenesis suppression (Efferth et al., 2001; Kelter et al., 2007; Geng et al., 2021). The therapeutic effects and mechanisms of ART in BLCA required further investigation. Network pharmacology was an innovative approach that integrated systems biology with multi-dimensional data integration techniques. This methodology facilitated the identification of various active components in traditional Chinese medicine and their potential targets. By constructing comprehensive drug-target-disease networks, network pharmacology systematically elucidated the intricate relationships between traditional Chinese medicine components and disease-associated targets. Molecular docking simulation was a computational technique used to predict the binding modes and interaction strengths between drug molecules and proteins. Using bioinformatics databases, this research identified potential core targets for ART in the treatment of BLCA. Molecular docking simulation effectively demonstrated the binding affinity and interaction sites between ART and its target genes. These simulations provided detailed insights into how ART interacted with these key proteins, revealing potential binding sites and the strength of these interactions, which were crucial for understanding its therapeutic effects. These predictions were subsequently validated through a series of experiments, providing insights into the mechanisms by which ART exerted its anti-BLCA effects. These findings revealed that ART exerted its anti-tumor effects through various mechanisms, including the induction of apoptosis, the disruption of cell cycle progression and the inhibition of the PI3K/AKT/mTOR signaling pathway.

In this study, molecular docking simulations revealed that ART formed a significant interaction with the apoptosis-related protein BCL-2, underscoring its potential role in regulating apoptotic pathways through this target. BCL-2 is an anti-apoptotic protein that plays a crucial role in cell survival by inhibiting apoptosis. It achieves this by preventing the release of cytochrome c from the mitochondria, which is a key step in the apoptotic cascade. By blocking this release, BCL-2 helps maintain cell integrity and viability, making it a critical factor in regulating programmed cell death. By blocking this release, BCL-2 inhibited the activation of caspases, which are critical executors of apoptosis (Ashkenazi et al., 2017). Based on flow cytometry analysis, it was evident that apoptosis levels in BLCA cells increased significantly with higher concentrations of ART. WB experiments revealed that ART reduced BCL-2 protein expression in BLCA cells, suggesting that ART promoted apoptosis by diminishing anti-apoptotic signaling. In contrast, Cleaved-Caspase 3 serves as a pivotal executioner in the apoptosis process. Once activated, this enzyme will initiate the cell death program by cleaving a range of cellular substrates. These cleavages lead to the dismantling of crucial cellular components, ultimately resulting in programmed cell death. The activation and function of Cleaved-Caspase 3 are essential for orchestrating the final stages of apoptosis, making it a key player in regulating cellular turnover and eliminating damaged cells. After ART treatment, the observed rise in Cleaved-Caspase 3 expression provided additional evidence that ART triggered apoptosis via the Caspase pathway. These findings suggested that the primary mechanism through which ART exerted its anti-tumor effects on BLCA cells is by inducing apoptosis.

Through network pharmacology analysis, this research identified that ART might exert its anti-BLCA effects by targeting the TP53 signaling pathway. Molecular docking simulations further confirmed that ART can effectively bind to the TP53 protein, suggesting that ART may activate this signaling pathway by directly acting on TP53. The TP53 gene encodes the p53 protein, a crucial tumor suppressor that primarily regulates cell cycle arrest, repairs DNA damage or initiates apoptotic mechanisms to prevent the proliferation of damaged cells. This function helps maintain genomic stability and prevents the accumulation of mutations. Under normal conditions, when DNA damage occurs, p53 is activated and functions as a transcription factor to regulate multiple target genes, thereby determining cell fate. If the damage is repairable, p53 induces cell cycle arrest, allowing sufficient time for DNA repair. However, if the damage is irreversible, p53 triggers apoptosis, promoting the clearance of damaged cells and preventing their proliferation (Wang et al., 2023). Additionally, p53 regulates the cell cycle by upregulating the expression of p21, a key checkpoint regulator. p21 inhibits the kinase activity of CDK4/6-CyclinD1 and CDK2-CyclinE complexes by directly binding to them, thereby preventing the transition from the G1 phase to the DNA synthesis phase (Engeland, 2022). This results in G0/G1 phase arrest, inhibiting cell proliferation and allowing sufficient time for DNA repair or other cellular responses. The G0/G1 phase is the initial stage of the cell cycle, during which cells grow, synthesize RNA and produce essential proteins for DNA replication. Therefore, upregulation of p21 not only effectively halts cell cycle progression but also ensures that cells have sufficient time to repair genomic damage, preventing the expansion and accumulation of mutated cells. Further experimental results confirmed that ART induced G1 phase cell cycle arrest in BLCA cells. Flow cytometry analysis showed a significant increase in the proportion of BLCA cells in the G1 phase after ART treatment, indicating that ART effectively blocked the transition from the G1 to the S phase. Additionally, WB experiments demonstrated that ART significantly reduced the expression of G1 phase markers CDK2 and CDK4 while increasing p53 expression, suggesting that ART promoted BLCA cell cycle arrest by activating the p53 pathway. CDK2 and CDK4 are key regulators of cell cycle progression and their activity are controlled by p53. Under ART treatment, the reduced expression levels of CDK2 and CDK4 further confirmed that ART inhibited these critical cell cycle proteins, leading to G1 phase arrest and effectively suppressing excessive proliferation of BLCA cells. In conclusion, this study demonstrated that ART exerted its anti-cancer effects by activating the p53 signaling pathway and inducing G1 phase arrest in BLCA.

In addition, this research utilized network pharmacology and molecular docking to identify that ART might act on AKT1, with a strong binding affinity for AKT1. It was hypothesized that ART directly bound to AKT1, thereby inhibiting its activity and exerting an anti-cancer effect in BLCA. The PI3K/AKT/mTOR signaling pathway is a core regulatory pathway involved in the malignant behavior of tumor cells, playing a critical role in tumor initiation and progression (Peng et al., 2022). Previous studies have shown that this pathway mediates various pro-cancer biological effects in tumor cells, primarily including the following aspects: (1) Promotion of cell cycle progression—by regulating key proteins involved in the G1/S phase transition, accelerating the progression of the cell cycle and thus promoting cell proliferation; (2) Inhibition of apoptosis—by suppressing both mitochondrial-mediated intrinsic apoptosis and death receptor-mediated extrinsic apoptosis signals, enhancing tumor cell survival; (3) Promotion of angiogenesis—by upregulating VEGF expression, promoting the survival, proliferation and migration of endothelial cells, inhibiting the expression of angiogenesis inhibitors and accelerating the formation of new blood vessels to support tumor growth (Yu et al., 2022; Deng et al., 2024). The PI3K/AKT/mTOR signaling pathway is a core regulatory axis in cell growth, proliferation, survival and metabolism, widely involved in the development and progression of various cancers. The activation of this pathway is usually triggered by the binding of growth factors or cytokines to their receptors (such as EGFR, PDGFR), which then activate PI3K to generate PIP3 and activate AKT. Activated AKT not only promotes the expression of cyclins (such as Cyclin D1) but also inhibits apoptosis-related proteins (such as BAX, FOXO), while activating mTOR to enhance cell proliferation and survival. As a downstream effector of this pathway, mTOR not only promotes protein synthesis and metabolic reprogramming but also enhances tumor cell tolerance and invasiveness by regulating HIF-1α (Fontana et al., 2024; Duan et al., 2018; Chen et al., 2024). Given the pivotal role of the PI3K/AKT/mTOR signaling pathway in cancer biology, inhibiting this pathway has become a key strategy in anti-cancer therapy. Further experimental validation indicated that ART could inhibit the phosphorylation activity of the PI3K/AKT/mTOR signaling pathway in BLCA cells, effectively suppressing tumor proliferation and survival. Experiments *in vivo* demonstrated that ART significantly delayed the growth of subcutaneous tumors in nude mice, further highlighting its potent anti-tumor effects through inhibition of the PI3K/AKT/mTOR signaling pathway. Moreover, ART not only effectively inhibited tumor progression but also showed favorable safety, indicating its potential as a promising therapeutic drug. As an inhibitor of the PI3K/AKT/mTOR signaling pathway, ART represents a promising therapeutic strategy that targets key mechanisms of tumor growth and survival. Future studies will explore the combination of ART with other therapeutic drugs to evaluate its potential synergistic effects, thereby further enhancing its efficacy and expanding its clinical applications.

In this research, it was also found that MYC might play an important role in the anti-cancer effect of ART on BLCA, and ART had a strong binding affinity for MYC. MYC is an oncogene belonging to the transcription factor family, mainly regulating key biological processes such as cell proliferation, metabolism, apoptosis and differentiation. The MYC family includes c-MYC, N-MYC and L-MYC, with c-MYC being the most extensively studied member. The expression regulation of the MYC is highly complex, mainly involving interactions with other transcription factors and regulatory factors to regulate the transcription of target genes, thereby participating in multiple physiological processes of cells. In normal cells, MYC expression typically supports cell growth, proliferation and differentiation. However, in tumor cells, the overexpression or uncontrolled activation of MYC often drives tumorigenesis and progression. The role of MYC in tumors manifests in several aspects, including promoting cell proliferation, metabolic reprogramming, inhibiting apoptosis, promoting genomic instability, modulating the tumor microenvironment and cross-regulating with other signaling pathways (Krenz et al., 2024). MYC-targeted WDR4 promoted proliferation, metastasis and sorafenib resistance by inducing CCNB1 translation in hepatocellular carcinoma (Xia et al., 2021). Therefore, MYC is not only a key driver of tumorigenesis but also a potential therapeutic target. Studies have shown that inhibiting MYC expression has significant anticancer effects in tumor treatment. Among these, the potential of natural compounds in inhibiting MYC expression has gradually gained attention. Licoflavone B suppressed myeloma growth by inhibiting MYC (Liu et al., 2022). Cryptotanshinone inhibited ovarian tumor growth and metastasis by degrading c-Myc and attenuating the FAK signaling pathway (Guo et al., 2022). Further research has found that ART inhibited aerobic glycolysis by suppressing c-Myc, thereby exerting anti-cancer effects in non-small cell lung cancer (Zhang et al., 2022). Thus, the application of natural compounds may emerge as a new direction for MYC-targeted therapy. Future research will focus on exploring how ART exerted its anti-cancer effects in BLCA through targeting the MYC, which will help better understand ART’s anti-cancer mechanism and provide a theoretical basis for developing new cancer treatment strategies.

## 5 Conclusion

In summary, this study combined network pharmacology and molecular docking to identify key targets of ART in BLCA and validated its therapeutic effects through experiments *in vitro* and *in vivo*. These findings provided a multi-dimensional mechanistic intervention strategy for the treatment of BLCA and offer important theoretical support for the development of targeted therapeutic strategies, laying the foundation for the further expansion of traditional Chinese medicine applications in BLCA treatment.

## Data Availability

The original contributions presented in the study are included in the article/Supplementary Material, further inquiries can be directed to the corresponding authors.

## Abbreviations

ART, artesunate; BLCA, bladder cancer; NMIBC, non-muscle-invasive bladder cancer; MIBC, muscle-invasive bladder cancer; PPI, protein-protein interaction.
